# Age-related dataset on the mechanical properties and collagen fibril structure of tendons from a murine model

**DOI:** 10.1038/sdata.2018.140

**Published:** 2018-07-24

**Authors:** Kheng Lim Goh, David F. Holmes, Yin Hui Lu, Karl E. Kadler, Peter P. Purslow

**Affiliations:** 1 School of Biological and Environmental Sciences, Stirling University, Stirling FK9 4LA, UK; 2 Manchester University, Wellcome Trust Centre for Cell Matrix Research, B.3016 Michael Smith Building, Faculty of Life Sciences, Oxford Road, Manchester M13 9PT, UK

**Keywords:** Ageing, Biophysics

## Abstract

Connective tissues such as tendon, ligament and skin are biological fibre composites comprising collagen fibrils reinforcing the weak proteoglycan-rich ground substance in extracellular matrix (ECM). One of the hallmarks of ageing of connective tissues is the progressive and irreversible change in the tissue mechanical properties; this is often attributed to the underlying changes to the collagen fibril structure. This dataset represents a comprehensive screen of the mechanical properties and collagen fibril structure of tendon from the tails of young to old (i.e. 1.6–35.3 month-old) C57BL6/B mice. The mechanical portion consists of the load-displacement data, as well as the derived tensile properties; the structure data consists of transmission electron micrographs of collagen fibril cross section, as well as the derived cross-sectional parameters. This dataset will allow other researchers to develop and demonstrate the utility of innovative multiscale models and approaches of the extra-cellular and physiological events of ageing of current interest to ageing research, by reducing the current reliance on conducting new mammalian experiments.

## Background & Summary

Aging is a complex multifactorial process underlying the accumulation of modifications to the molecules in the cells and extracellular matrix of the body^1^, leading to cellular senescence^2^, and consequential pathological degeneration of normal function^3^. Connective tissues, such as tendon, can be considered as biological fibre reinforced composites with collagen responsible for reinforcing the weak proteoglycan-rich ground substance in extracellular matrix (ECM)^4^. Collagen also forms the basis for the tissue hierarchical multiscale arrangement^5,6^. The tendon hierarchical structure is well-known: from the nanometer to centimeter length scale, one finds rod-like collagen molecules, sub-fibrils, fibrils, fibres (bundles of fibrils), fascicles (bundles of fibres) and finally the whole tissue. With regards to the ageing process, the progressive and irreversible change in the mechanical properties of tendon has been a subject of numerous studies^7^, e.g. animal model studies^8,9^, motivated by the need to understand tissue homeostasis^10^, and how age-related changes in ECM composition may result in tendons susceptibility to tendinopathy in old individuals^9^. Clinical studies have also seen an increase in tendon degeneration or rupture with age^11,12^.

The reinforcement of tendon ECM by collagen fibrils results in the tissue having high toughness and strength^13^. These functional properties are influenced by the structure, including composition, of the collagen fibrils^13,14^, parallel to current understanding on the influence of fibre shape, size and material on the composite mechanical properties^4^. As the individual ages, age-related changes to the mechanical function are accompanied by changes to the structure of the collagen fibril^15,16^. Others have pointed out that this relationship between collagen structure and tendon mechanics could be confounded by the inherent effect of the reduction of small leucine-rich proteoglycans such as biglycan^17^. Above all, the key challenge to understanding the age-related changes in the structure-function relationship lies in identifying and quantifying the structure at length scales corresponding to collagen fibrils^13^. The conventional arguments are that fibrils with large diameters (Ds), which are observed in tendon with increasing age, may inherit a high density of intrafibrillar covalent cross-links and this could regulate tensile fracture resistance mechanics^18,19^. On the other hand, fibrils with small Ds may play a role in creep resistance mechanics because the smaller cross-sectional dimension could implicate higher surface area per unit mass of the fibrils and, in turn, higher density of noncovalent cross-links between the fibrils and the interfibrillar matrix components^18,19^. The main issues with the D parameter are: (1) histogram profiles of the D distribution in many tissues are typically non-Gaussian ^8^, mainly unimodal or bimodal depending on the age and tissue type^17,20^, (2) a spread of values, narrow in foetal tissues^20^ but wide in adulthood^16,18^, (3) an increasing irregular shape in the fibril cross section with age, attributing to fibril-fibril fusion^21–23^. These issues suggest that the diameter parameter could mask the true extent of the variability if the dataset is reduced to a single mean value.

As part of the EU framework V project on 'Mechanisms of Ageing in ECM', we have reported evidence of the relation of the mechanical properties of tendon, namely tensile strength and stiffness, to the fibril cross-sectional area fraction (ρ; related to the fibril volume fraction), in the presence of ageing, according to the simple rule of mixtures for fibre composites^18^. The dataset comprises the mechanical portion, namely the load-deformation data, and the structural portion, namely transmission electron micrographs of fibril cross sections^18^. This dataset is also the starting point for the re-analysis of data necessary for modelling how age-related variations in tendon resilience and resistance to rupture were directed by subtle changes in the bimodal distribution of D in a subsequent study^19^.

In recent years, powerful computers with processors capable of implementing deep networks have been deployed successfully to evaluate datasets for identifying complex and subtle patterns in data^24^. With regards to potential reuse value, this would be consistent with the reduction argument in the principles of 3Rs (i.e. replacement, refinement, reduction) for animal use^25^. This dataset will be invaluable for investigators who have developed (or plan to do so) innovative computational multiscale models and approaches for ageing research^26^—the validation and utility of the models and approaches can be demonstrated through this dataset rather than relying on data derived from new mammalian experiments^25,27^.

## Methods

In this section, we describe the procedures used in producing the mechanical and structural data. This description covers the experimental design from the aging of animals, acquisition of tissues from the animals at the respective age groups and to the mechanical testing and electron microscopy. The methods addressed in this section are expanded versions of descriptions in our related work^18,19^.

### Experimental flowchart

**Figure 1:**
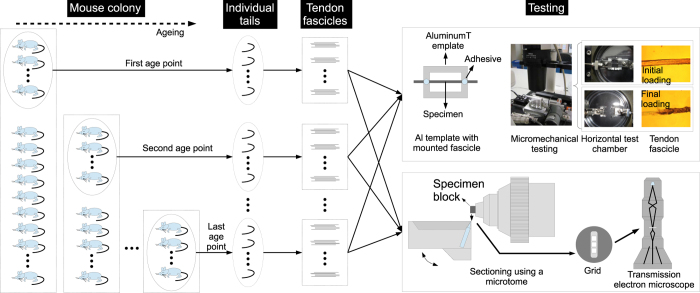
Experimental design of the structure-mechanical property study. The experimental workflow is depicted from left to right. A young mouse colony was established at Manchester University. The mice were culled before transporting them to Stirling University for tail tendon fascicle harvesting, followed by micromechanical testing, and finally, data processing to determine the mechanical properties. The age groups (and the tail sample size, N) are as follows: 1.6 month-old (N=3), 2.6 month-old (N=3), 4.0 month-old (N=3), 11.5 month-old (N=4), 23.0 month-old (N=3), 29.0 month-old (N=4), 31.5 month-old (N=4) and 35.3 month-old (N=4). For each tail, several tendon fascicles (technical replicates) segments were prepared and mechanically tested using a micromechanical tester, to rupture. A portion of the fascicles prepared for mechanical testing were used for imaging by transmission electron microscopy (TEM) to digitally capture the cross-section of the fascicle, at Manchester University.

Figure 1 shows a schematic of the experimental design for the study of the structure-mechanical property resulting in the dataset for the load-displacement response curves, the collagen fibril diameter and collagen fibril area fraction. The experimental design involves establishing a mouse colony and ageing the colony to predetermined age points, tail dissection after culling, tendon fascicles harvesting from the tail, mechanical testing to rupture to investigate the micromechanical properties and imaging by transmission electron microscopy (TEM) to investigate the collagen fibril structure.

**Figure 2:**
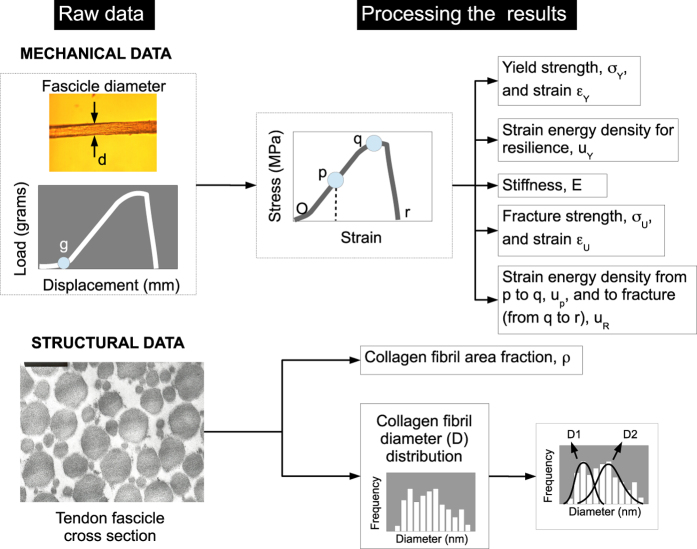
Generation of derived data. With regards to the mechanical data, after obtaining the mechanical data, namely the load-displacement curves and the corresponding fascicle diameter, d, and grip-to-grip distance, L_0_, these were used to derive the curve in the stress versus strain graph. (Here, O, p, q and r represent the origin, point of inflexion, maximum stress and rupture point, respectively.) Thereafter, from the stress-strain curve, the respective mechanical properties are determined. With regards to the fibril structural data, after obtaining the TEM images of the fascicle cross sections, the images were analysed to determine the collagen fibril area fraction, ρ, and the histogram of the frequency of collagen fibrils versus fibril diameter, D. From the frequency-D data, further analysis was carried out to determine the mean D, namely D_D1_ and D_D2_, of the respective fibril sub-populations, D1 and D2.

After mechanical testing and imaging by TEM, the results were processed. Figure 2 shows a schematic of the generation of the data for the mechanical and structural properties for each fascicle specimen. The mechanical properties comprised, namely, the yield strength, σ_Y_, and the corresponding yield strain, ε_Y_, and stiffness, E (at point p) fracture strength, σ_U_ (together with the strain at σ_U_, denoted by ε_U_, at point q), as well as the strain energy densities for resilience, u_Y_ (from the origin O to point p), plasticity, u_P_ (from point p to q), and fracture, u_R_ (from q to r). The structural properties comprised the collagen fibril area fraction, ρ, and the collagen fibril diameter, D.

### Mouse colony

All work with regards to the use of the mouse tissue was approved by and performed in accordance with the guidelines from the ethical committees related to the institutions, namely Manchester University and Stirling University, where the project was based.

A young (1.6 month-old) C57BL6/B male mouse colony was established in Manchester University, in compliance with a licence from the UK Home Office under section of the Cruelty to Animals Act. The mice were housed in the animal care facility according to IACUC approved protocols. Subsequently, the mice were randomly picked and sacrificed at predetermined age points. All the mice used in this study appeared normal; they did not show any observable pathological signs. The total number of mice used for this study was 28. The mouse replicates for the respective age groups are indicated in Table 1.

### Fascicle extractions

To harvest the fascicles, the mice were anesthetized and sacrificed in accordance with guidelines laid down by the Home Office, UK, for the care and use of laboratory animals. The tail was severed from the body using a surgical blade. By making an incision on the skin with a surgical blade, at the base of the tail, in the direction along the tail axis, the skin could be easily removed with the help of two pair of fine tweezers. This exposed the tendon fascicles; each fascicle could be removed by sliding them out along the tail using a pair of fine tweezers, with minimum force to avoid damaging the fascicles. A selection process was applied to the fascicles by examining each fascicle under a microscope for possible damaged features, e.g. badly frayed fibril bundles; the fascicle was discarded if damage was suspected. The specimens were then stored in a freezer at −20 °C until needed; freezing at −20 °C is not known to cause appreciable alteration to the mechanical properties^28,29^.

### Micromechanical tests

A custom-made small-scale horizontal tensile test frame device (Fig. 1) was used to stretch the tendon to rupture. The device comprised: (1) a force transducer (450 g linear range, model UF1, Pioden Controller Ltd., Canterbury, UK) to measure the load generated in the tissue during the process of stretching, (2) a linear variable differential transformer (type 5D/500 A, RDP Electronics Ltd., Wolverhampton, UK) to measure the grip-to-grip tendon sample displacement as the specimen undergoes deformation, and (3) a DC motor to stretch the sample at a controlled (crosshead) displacement rate. The use of the horizontal test frame enabled individual specimen to be tested in a hydrated state, throughout the experiment, by submerging it in phosphate buffer saline (pH 7.2) in a Petri dish that was fitted within the frame. This was intended to mimic the hydrated physiological condition in the tail.

The method for preparing a fascicle specimen for testing was outlined in previous paper^30^. This method is elaborated here for information. To prepare a specimen for testing, the specimen first had to be mounted across a rectangular aluminium frame (known as the 'template'; Fig. 1). Cyanoacrylate adhesive was used to secure the specimen on the template; a toothpick was used to collect a minute amount of the adhesive (by dipping the sharp end of the toothpick into a reservoir of the adhesive) and to transfer the adhesive onto a predetermined point at the surface of the template border; a minute amount of the adhesive was applied to a second point but on the opposite border of the template. Second, fascicle specimens of length 7 mm were prepared by sectioning the extracted fascicle using a surgical blade. The sectioned specimen was then laid across the template where its ends contacted the adhesive (Fig. 1). Thereafter the template was mounted on the grips of the tester. It must be emphasized that the specimen-template technique (1) facilitated the alignment of the fascicle axis in the direction of the displacement on the tensile device and (2) minimized undue stretching of sample prior to testing^30^. Finally, the borders of the template adjacent to the fascicle specimen were severed using a pair of scissors prior to the start of tensile testing.

The rig was mounted on the stage of an inverted microscope (Zeiss Axiovert 25). The thickness, d, of the fascicle specimen (Fig. 2) was recorded under the microscope at six different locations along the axis of the specimen. These values were then averaged to obtain the mean thickness. Assuming a circular profile for the cross section of the fascicle, under this circumstance, d was used to estimate the diameter of the tendon fascicle; the fascicle cross-sectional area, A, was set equal to πd^2^/4.

Each fascicle specimen was stretched from a slightly slackened state until rupture, at a predetermined displacement rate of 0.067 mm/s. This value was implemented for consistency with previous studies reported by other research groups^15,31^. The reason for stretching from slack to taut was to allow for the determination of the point where stretching started; this point was used to define the origin of the load and displacement data. Raw data (i.e. load and grip-to-grip distance) resulting from this method is contained in tab-delimited text files (mtt01.zip to mtt35.zip, Data Citation 1). Thereafter, the raw data was processed using a zeroing procedure (see first and second paragraphs of section Determination of the mechanical properties) to obtain the load-displacement data and subsequently the stress versus strain data. The strain parameter was set equal to the ratio of the displacement to the sample nominal length.

### Determination of the mechanical properties

An algorithm, strainenergy_v4_1.m (version 4.1), was developed to evaluate the load-displacement data^32^. The algorithm was implemented using Matlab (version 6; MathWorks, Natick, MA). The load-displacement data were read and processed from the tab-limited text file, using the following input parameters, fascicle diameter, last strain point, order of polynomial, 'load-to-start' at 1% of maximum load. The 'load-to-start' parameter required a Boolean input, namely 'y’, i.e. yes, or 'n', i.e. no, as part of the 'zeroing' the load and displacement data procedure. In this procedure, first the maximum load was determined. Next, the starting load was zeroed; the zero-load point may be determined by justifying for the load to start at 1% of the maximum load (i.e. 'y'); otherwise no action was needed (i.e. 'n'). This semi-automated approach was used to cut off the initial data that described the slack in the specimen before it became taut (point g, Fig. 2), i.e. when the deforming specimen straightened from a slacken state to a taut state, using a load-cutoff value equal to 1% of the maximum load. The extent of the cut-off region was also checked by eye, i.e. by comparing the stress-strain curve to the original load-displacement curve, for further confirmation. In some cases, the presence of noise at low loads made it difficult to execute the semi-automated approach. Consequently, a manual approach was applied to truncate the initial data points before running the strainenergy_v4_1.m to evaluate the load-displacement data further. (In this case, the 'load-to-start' was set='n'.) The specimen nominal length, L_0_, was set equal to the displacement at zero-load point; thereafter, to complete the zeroing procedure the displacement was set equal to zero and the origin of the load-displacement graph was set to the zero-load point.

After the zeroing procedure, the load-displacement data was converted to stress-strain data. The origin of the load-displacement graph corresponded to the origin of the stress-strain graph (point O, Fig. 2). The corresponding stress (σ) in the specimen at a given displacement was determined by the ratio of P to A: that is σ=P/A. The corresponding strain (ε) in the specimen at a given displacement was determined by the ratio of displacement, x, to L_0_: that is ε=x/L_0_. In all cases, the stress-strain curves featured profiles that were typical of tail tendons described in reports from other research groups^15,31^.

The mechanical properties for each fascicle specimen were determined as follows. The strain energy density parameters, namely resilience (u_Y_, from O to p, Fig. 2), plastic loading (u_P_, from p to q, Fig. 2), rupture (u_R_, from q to r, Fig. 2), and u_F_ (=u_P_+u_R_), were determined numerically using the trapezium rule. The maximum stress, i.e. at point q, corresponded to the strength, σ_U_; the strain at maximum stress was denoted by ε_U_. The stress and strain at point p corresponded to the yield stress (σ_Y_) and yield strain (ε_Y_), respectively. The gradient at the point of inflexion, p (Fig. 2), corresponded to the stiffness, E; this approach was consistent with that used in reports from other research groups^15,31^ and represented the maximum modulus observed. To determine the point p, the fitting of the polynomial curve to the stress-strain data from the origin to the maximum stress was implemented. Using the finite difference approach, the point p was determined by plotting the dσ/dε versus ε and evaluating the maximum dσ/dε within the region of the stress-strain curve from the origin to the point (point q, Fig. 2) at maximum stress.

The respective mechanical property of each individual tail was determined by averaging the mechanical property derived from each fascicle specimen (i.e. the technical replicate). The respective mechanical property corresponding to each age group was determined by averaging the mechanical property derived from each tail. Data resulting from this method can be found in a MS Excel file (mechprop.xls, Data Citation 1).

### Transmission electron microscopy

Tendons were fixed in 2% glutaraldehyde in 100 mM phosphate buffer, pH 7.0, for 30 min at 20 °C, followed further by 2 h at 4 °C. After washing in 200 mM phosphate buffer, the samples were fixed in 1% glutaraldehyde and 1% OsO4 in 50 mM phosphate buffer, pH 6.2, for 40 min at 4 °C. After rinsing with distilled water, they were stained with 2% aqueous uranyl acetate en bloc for 16 h at 4 °C, dehydrated in ethanol and embedded in Spurr resin. An ultramicrotome was used to prepare ultrathin sections (about 70 nm thick) of the specimens (Fig. 1). Each section was mounted onto a grid to allow for examination in a TEM (Tecnai BioTwin instrument, FEI, Eindhoven, Netherlands). An accelerating voltage of 80 kV was set for collecting the micrographs. Magnification of the TEM was calibrated using a diffraction grating replica (2160 mm^−1^). TEM images (Table 1) of near-transverse sections (i.e. with all fibrils in a near-transverse section) of the fascicles were digitized from photographic film acquired at an instrumental magnification of x15 000. TEM images of equal-sized (4×5 μm^2^) fields were obtained. To achieve a representative sampling of collagen fibrils in the fascicle tissue of each mouse, for the selection of the regions of interest for image sampling when scanning over the cross-section of a fascicle we adopted the following criteria established in other studies^31^: (a) only well-defined cross-sectional regions, i.e., the regions which were sheathed by a paratenon layer, were used; (b) the region of the peripheral paratenon was not used; (c) if an area overlapped with a grid bar, or included a cell, a tear or a fold, or a poorly stained region where it was not possible to achieve a sharp focus, then the adjacent area was selected; (d) images were obtained from different fascicles within a tail. The digital images resulting from this method can be found in tif formatted file (Scan001-046.zip to Scan169-192.zip, Data Citation 1).

### Determination of the structural properties

The TEM images were used to measure D (fibril diameter) and ρ (fibril cross-sectional area fraction) using commercial image analysis software (SEMPER5, Synoptics, Cambridge, UK). To minimise the risk of selection bias, for each age group, a randomisation approach was implemented to obtained a sample of N_c_ images (i.e. technical replicates) derived from the pool of images, where subscript c denotes the age group id (1,2,...,35). (The pool of images was established after a survey was carried out over widely separate locations across individual fascicles of that age group as pointed out in the section Transmission electron microscopy.). Firstly, images from the image pool were randomly sorted by tagging a randomly generated number (between 0 and 1) to the image file name, and then performing a sorting process to order the random numbers starting from the smallest to the largest. Secondly, the value for N_c_ was randomly derived from a range of integers, namely 4-11. Then N_c_ images were selected from the sorted images by identifying the first N_c_ images. For each age group, images in each animal were then combined and considered representative of the collagen fibril profile in fascicles from that age group^31^. From the combined images, structural parameters related to ρ and D were determined^31^. The ρ value was evaluated by averaging the ρ determined from the N_c_ images. To determine ρ, the cross section of each fibril (or part fibril at the image boundary) was manually traced, and the area, a_f_, of individual fibril was computed using the image analysis package. The value of ρ was computed as the ratio of the sum of a_f_s values to the total sample area^33^. For the measurement of D, following a common approach that has been reported elsewhere^31,34^ D was derived from the a_f_ by modelling the shape of the fibril as circular. We set a_f_ equal to πD^2^/4.

The algorithm used to evaluate the mean Ds of the collagen fibril subpopulations is found in fibriprogram_v5.m^35^ (version 5). This approach has been reported in an earlier paper^36^. For simplicity, the approach assumed the smallest number of subpopulations to model non-Gaussian profiles of the frequency histogram (i.e. the primary distribution). In all cases, the smallest number was found to be two, and we shall refer to these subpopulations by D1 and D2 for simplicity (Fig. 2), where the mean D associated with D1 (i.e. D_D1_) is smaller than the mean D associated D2 (i.e. D_D2_). An approach based on the finite mixture modelling, complemented by an optimisation strategy known as the simulated annealing (SA) approach, was implemented to evaluate the mean Ds of the sub-populations.

To determine the optimal solution to the mean and SD of D for the respective subpopulations, the SA optimization approach evaluated for possible frequency-D profiles that best fit the primary distribution. To execute the SA approach, the “temperature” parameter was assigned a value of 0.5 with a reduction factor of 0.9. During each run, the maximum number of configurations that the SA algorithm could explore was fixed at 100; the number of temperature steps to be executed was fixed at 100; and the number of successes allowable before looping to the next temperature steps was fixed at 10. The configuration space addressed the fibril subpopulations D1 and D2 (Fig. 2). The Gaussian profile of these subpopulations was defined by the amplitude-which described the proportion of fibrils in the subpopulation-mean and SD; the magnitudes of these parameters were assigned from a predefined range of values using an algorithm for randomizing the selection of values. An initial run was executed to obtain a preliminary range of values for the mean (i.e. D_D1_ and D_D2_) and the associated SD, respectively, followed by a refinement run by narrowing the range of values. Each set of configuration parameters was evaluated by a linear regression algorithm, which was executed to fit the profiles of D1 and D2 to the primary distribution (Fig. 2).

Data resulting from this method is can be found in a MS Excel file (strucprop.xls, Data Citation 1).

### Code availability

The code, strainenergy_v4_1.m, for generating and processing the dataset for load-displacement and stress-strain is publicly available through Figshare^32^. Software Matlab (version 6; MathWorks, Natick, MA) was used to run the code^18^. The specific variables of the parameters used to generate the current dataset are as follows:

- ip1: input file containing the load-displacement data
- diameter: fascicle diameter
- laststrainpt: an estimate of the strain at rupture (r, Fig. 2)
- orderpoly: an integral value from 2–7 which represents the order of the polynomial for fitting to the data from O to q (Fig. 2)
- loadat1percent: y/n; to determine the value of the load-to-start (set at 1% of the maximum load) at which the specimen became taut. ‘y’ denotes yes; ‘n’ denotes no.

The 'log file', logfile.txt, contains the parameters used for deriving the values of the respective mechanical properties (namely σ_Y_, ε_Y_, u_Y_, E, σ_U_, ε_U_, u_P_, u_R_) and is distributed as part of the strainenergy_v4_1.m code^32^. All codes are internally documented to explain their purpose; this would also be useful for users who should wish to customize them.

The code, fibriprogram_v5.m, manages the optimisation process for fitting the Gaussian curves to the frequency versus D dataset by calling on sub-routines generation of a Gaussian function (gaussiansingle_v5.m), linear combination of the Gaussian functions (gaussiancomposite_v5.m), SA (simulanneal_v5.m) and the metropolis rule (metrop_v1.m). These m files are publicly available through Figshare^35^. Software Matlab (version 7; MathWorks, Natick, MA, backward compatible to version 6) was used to run the code^19^. To execute the SA simulation for the respective age group, the main program, fibriprogram_v5.m, reads an input parameter-cum-data file (fibrilprogram_cM_v5.m, where c denotes the age group id, namely 1, 2,...35) containing input values of the following parameters:

- NCONFIG: number of configurations
- NSUCCLIMIT: the number of successes allowable before looping to the next temperature step
- NTRIALS: the number of temperature steps to be executed
- SEED: a seed value for starting the random number generation
- Temperature: the annealing temperature parameter
- Tfactr: reduction factor for temperature
- LOWER UPPER:LIMITS OF FIB DIAM
- ngauss: number of Gaussian distributions; in this study, ngauss=2
- SCALE FACTRS:INITIAL GUESS
- mu(i), sigma(i): mu and sigma represent the respective mean and standard deviation of the ith Gaussian distribution, where i=1 to ngauss
- MUA(i) MUB(i): MUA and MUB are 'amplification' coefficients for the generation of random numbers for the i^th^ mu
- SDA(i) SDB(i): SDA and SDB are 'amplification' coefficients for the generation of random numbers for the ith sigma
- FIBRIL_SIZE FREQUENCY: data of the frequency versus fibril diameter, D (of the respective age group), available from a MS Excel file (strucprop.xls, Data Citation 1)

The input parameter-cum-data files are found in an archived file, fibrilprogram_cM_v5.zip, distributed as part of the fibriprogram_v5.m code^35^. All codes are internally documented to explain their purpose; this would also be useful for users who should wish to customize them.

The results generated by fibriprogram_v5.m were the mean values, D_D1_ and D_D2_, of the respective sub-populations, D1 and D2, and a plot of the respective Gaussian distributions overlaying the primary distribution of the frequency-D histogram (Fig. 2).

## Data Records

All data records described in this manuscript are available in the Figshare repository (Data Citation 1), organized according to the respective data modality. The format, content and availability of the depositions are described in the following subsections and in Table 1.

### Data record 1—The fascicle load-displacement data

The quantitative data relating to the fascicle load-displacement characteristics are recorded in tab-delimited text files (mtt01.zip to mtt35.zip, Data Citation 1). These files are organized by age groups and archived with the following names mtt01.zip, mtt02.zip, mtt04, mtt11.zip, mtt23.zip, mtt29.zip, mtt31.zip and mtt35.zip (Table 1). The individual tab-delimited text files are named as mttNN_X_tYZ.txt, where NN corresponds to the age group id, X denotes the sample id, t represents tag, Y represents the fascicle number, Z (a letter) represents the specimen derived from fascicle number Y. These text files contain data derived from the respective fascicle segments (i.e. technical replicates).

The column descriptions for the data in the respective text files are as follows:

COLUMN A—labelled as 'Time' (ms); the time point corresponding to the load-displacement data point

COLUMN B—labelled as 'Displacement'; signal (mV) corresponding to the grip-to-grip displacement inclusive of the initial distance between the grips;

COLUMN C—labelled as 'Load'; signal (mV) corresponding to the load in the specimen, as detected by the load-cell;

COLUMN D—also labelled as 'mm'; displacement data (mm)

COLUMN E—also labelled as 'grams'; load data (mm)

### Data record 2—The fascicle mechanical properties

The quantitative data relating to the fascicle diameter, d, measurements of the respective specimen in each age group are contained in a worksheet (labelled as 'Fascicle diameter' in mechprop.xls, Data Citation 1). The column descriptions for this worksheet are as follows:

COLUMN A—Age group

COLUMN B—Samples

COLUMN C—File name of the individual fascicle specimen

COLUMN D—Fascicle diameter (μm), a mean value from 6 repeated measurements

COLUMN E—Standard error of the mean (μm),

The quantitative data relating to the derived fascicle mechanical properties are found three worksheets, namely 'Derived parameters', 'Mean-individual mouse' and 'Mean--age group' in mechprop.xls. The column descriptions for the worksheet 'Derived parameters' are as follows:

COLUMN A—Age group

COLUMN B—Samples

COLUMN C—Input file names; for these names, 'mtt' represents mouse tail tendon.

COLUMN D—Stress at the point of inflexion, sY (MPa)

COLUMN E—Strain at the point of inflexion, eY

COLUMN F—Stiffness at the point of inflexion, E (MPa)

COLUMN G—Maximum stress, sU (MPa)

COLUMN H—Strain at maximum stress, eU

COLUMN I—Strain energy density to resilience (from origin to point of inflexion), uY (MPa)

COLUMN J—Strain energy density during plastic loading, from point of inflexion to maximum stress, uP (MPa)

COLUMN K—Strain energy density during rupture, from point of maximum stress to point of rupture, uR (MPa)

COLUMN L— Strain energy density during failure, from point of inflexion to rupture, uF (MPa)

COLUMN M—Total strain energy density from origin to rupture, u0 (MPa)

COLUMN N—uY/sY

COLUMN O—uF/sU

The column descriptions for the worksheet 'Mean-individual mouse' are as follows:

COLUMN A—Age group

COLUMN B—Samples

COLUMN C to L— sY, sU, E,uY, uP, uR, uF, u0, uY/sY, uF/sU

The column descriptions for the worksheet ''Mean- age group' are as follows:

COLUMN A—Age group

COLUMN B to K—Mean sY, sU, E, uY, uP, uR, uF, u0, uY/sY, uF/sU

COLUMN L to U—Standard error of mean sY, sU, E, uY, uP, uR, uF, u0, uY/sY, uF/sU

### Data record 3—The TEM images

The TEM images (TIF format) of the cross sections of fascicles segments (technical replicates) from the C57BL6 mouse tail tendons of the respective age groups are named as ScanNNN.tif, where NNN=001, 002, 003,... 191,192, organized in 8 archived files (Scan001-046.zip to Scan169-192.zip, Data Citation 1). A description of the respective image files is provided in a table, found in a MSWord file (TEMtable.doc, Data Citation 1).

### Data record 4—The collagen fibril structural properties

The quantitative data relating to the derived fibril area fraction from TEM images of the respective age groups are contained in the worksheet (labelled 'Fibril area fraction', in strucprop.xls, Data Citation 1). The column descriptions for the worksheet, i.e. COLUMN A to H, contain the fibril area fraction of fascicle specimens derived from the individual mouse of the respective age groups namely 1.6, 2.6, 4.0, 11.5, 23.0, 29.0 31.5 and 35.3 month-old. COLUMN J, K and L respectively summarized the datasets with descriptive statistics for each age group, namely the mean of the fibril area fraction and the corresponding standard error of the mean.

The quantitative data relating to the derived fibril diameter from TEM images of the respective age groups are contained in the worksheet, 'Fibril diameter', in strucprop.xls. The column descriptions for the worksheet are as follows

COLUMN A—Fibril diameter interval (nm), 'bin' for the histogram plot

COLUMN B to I—Frequency of the corresponding fibril diameter interval, for the respective age groups, namely 1.6, 2.6, 4.0, 11.5, 23.0, 29.0 31.5 and 35.3 month-old.

COLUMN K to R—Normalized frequency of the corresponding fibril diameter interval, for the respective age groups, namely 1.6, 2.6, 4.0, 11.5, 23.0, 29.0 31.5 and 35.3 month-old.

COLUMN T to X—Age groups and the respective (mean and SD) D of D1 and D2

## Technical Validation

The experimental design presented in this dataset has been validated in several ways. First, the mechanical tests, complemented by examination of the video images taken under the microscope, were performed at different displacement rates on (1) tissue fascicles from a different animal model^37^ and (2) synthetic biopolymer-based microfibre^38^ to ensure that the sensitivity of the tester yielded results to consistency with findings attributing to the strain-rate dependence of a viscoelastic material. Mechanical tests were also carried out on the synthetic biopolymer-based microfibre to ensure that the tester sensitivity yielded results to consistency with findings attributing to the load generated from specimen of varying sizes. Second, the mean values of the derived mechanical and structural parameters, as well as the degree of variability, were compared with previous studies reported for the corresponding age point^31^ to enable us to make a decision on the acceptability of the results.

For the validation of the statistical models, with regards to the simple linear regression analysis, the respective mechanical properties, namely σ_U_ and E versus ρ, were tested for normality and homogeneity of residuals. The normality plot revealed residuals distributed somewhat uniformly about a central peak but with little sign of skewness. The homogeneity of residuals plot revealed a somewhat well-dispersed residuals about the zero line, suggesting little sign of inhomogeneity in the distribution of points about the zero line^18^. With regards to the multiple (linear) regression analysis, the respective mechanical properties, namely u_Y_/σ_Y_ (or u_F_/σ_U_) versus D_D1_ and D_D2_, were also tested for normality and homogeneity of residuals. Similarly, the normality plot revealed no appreciable sign of skewness; the homogeneity of residuals plot revealed a homogeneous distribution of points about the zero line^19^. Multicollinearity of D_D1_ versus D_D2_ was assessed using the Pearson correlation coefficient test. We reported that the Pearson correlation coefficient=0.599 (ref. 19); we concluded that the D_D1_ and D_D2_ were not correlated with each other, accompanied by a cautionary note that the Pearson correlation coefficient was marginally less than the tolerable threshold, i.e., 0.600 (ref. 19).

For informational purpose, we summarized the datasets with descriptive statistics as follows in Table 2.

With regards to the protocol for the selection of TEM images, the overall purpose of the randomisation approach, which involves a random selection of the acquired images according to the (random) number of samples of the respective age group, is to reduce the risk of selection bias so as to achieve a set of representative images for determining the area fraction and histogram of frequency versus fibril diameter for each age group. As described in the section Transmission Electron Microscopy, we have followed a strict set of criteria, adopted from the report of Derwin and co-worker^31^ for surveying and acquiring TEM images; the final numbers (N_c_s) of TEM images acquired varied from 12 to 42. While the N_c_s were not as well-balanced across all age groups as we might wish, if one adopts a single-stage randomisation approach by randomly recruiting the individual images from each age group such that the N_c_ satisfy a predetermined number that forces the number of images in each age group to be the same (the clearest choice of this number would be equal to the age group with smallest number available), this could result in the dependence of the final result on the limited number of images available in those age groups. Alternatively, an additional stage was implemented by requiring a random-number of samples to be assigned to N_c_ of each age group. In retrospect, the results showing (1) the increase in collagen area fraction with age (in young mice, from 1.6 to 4 month-old) and the lack of an appreciable change from maturation to old age, and (2) the shifting of fibril diameter distribution from the region of smaller size (in young mice) to larger size (in mature and old mice) thereafter, are not surprising as these are consistent with findings reported in other tissues, e.g. rat tail tendons^39^. Given the imaging constraints that we have described above, we believe that there is value in using such an approach to achieve a set of representative images for determining the area fraction and histogram of frequency versus fibril diameter for each age group.

This paragraph is concerned with a technical discussion of the power of the tests that we have performed. This involved the prospective study to consider design sensitivity before the project started, and the retrospective study to understand the power of tests that we have already conducted. The prospective study considered the results of the stiffness (E) and strength (σ_U_) of the mouse tail tendons from 3 and 8 week-old mice, respectively, based on the report published by Derwin and Soslowsky^31^, where a sample size of N=6 per age group was used. To the best of our knowledge, the Derwin and Soslowksy report^31^ was the only relevant literature available at that time for mouse tail tendons. The power analysis was carried out on the E and σ_U_ values using the means and SDs derived from the 3 versus 8 week-old mice. In all cases, the analysis predicted a power of one. Additionally, the analysis predicted that the power equals 0.99 when the number of mice per age group was set to 3. Is the sample size of mice per age group in our study sufficient to adequately address the age-related changes in the mechanical properties research question? In the retrospective study of our power analysis, for the purpose of illustration, with regards to the σ_U_ of the 11 month-old age group versus the 1.6 month-old age group, a two-sample t-test (for unequal sample size and variance) power analysis yielded a power equal to 0.98 for a two-sided case. If one expects that the σ_U_ of the 11 month-old age group to be greater than the younger age group—as in the case of rat tail tendons^40,41^—the (one-sided) power increased to 1.0. To continue this technical discussion, it is noted that the E for the 11 month-old age group versus 1.6 month-old age group yielded a (two-sided) power equal to 0.57. If one expects the E of the 11 month-old age group to be greater than that of the younger age group—as in the case of rat tail tendons^41^—the (one-sided) power increased to 0.73. Nevertheless, both cases pointed to a low likelihood of not detecting an age-related difference in E when the difference does actually exist. To further illustrate the age-related difference in E, it is noted that a comparison of E for the 4 month-old age group (that is characterised by a lower mean as well as smaller SEM, i.e. higher precision of the estimate of the mean, see Table 2) with that for the 1.6 month age group yielded powers of 0.85 (two-sided) and 0.94 (one-sided). With regards to these instances, we recommend interpreting these finding conservatively given that a lower power (<0.8) for the E was observed in the older age groups (e.g. 11 month-old) versus the younger age group (namely 1.6 month old). Although this study was prompted by observations of the relationship between structure and mechanical properties in the presence of ageing in the tendons from young mice^31^, we believe the result presented here provides first-hand information for future tests to modify the experimental design to increase the power and continue to evaluate the same problem or address research questions other than those it was created for.

This paragraph is concerned with a technical discussion on estimating the bias error. For the mechanical testing of the fascicles, an uncertainty arose from the measurements carried out during the calibration of the load cell and the displacement transducer. The magnitudes of the uncertainty in the fascicle displacement measurement, fascicle thickness measurement (d) and load cell readings are estimated to be 0.01 mm, 0.0025 mm and 0.0001 grams, respectively. We predicted the propagation of these uncertainties to the uncertainty of the respective derived quantities using simple add-in-quadrature models. We recall that uncertainty, δQ, of a quantity, Q, derived from the addition (or subtraction) of quantities a and b is estimated as δQ=√([δa]^2^+[δb]^2^); where this involves the multiplication (or division) of a and b, we used δQ=Q√([δa/a]^2^+[δb/b]^2^). For the purpose of illustration, we will consider the measurements associated with the 4 month-old age group. To estimate the uncertainty (δε) in the strain ε, we note that the length of the fascicle L_0_ is of order of magnitude 5 mm. Consider a fascicle fracturing at a displacement of 1.00 mm with a corresponding maximum ε=0.2; numerically, we find that δε=0.002. The extensibility at maximum stress for the 4 month-old age group has a mean value equal to 0.060 with an estimated precision (i.e. SEM) of 0.005 (mechprop.xls, Data Citation 1), which is 2.5 times larger than the δε. To estimate the uncertainty (δσ) in the stress σ, consider (1) a fascicle with d=0.1 mm, and a corresponding cross-sectional area, A, equal to πd^2^/4=8x10^-3^ mm^2^ (with an uncertainty δA=3×10^-4^ mm^2^), and (2) a load of 100 grams is applied to fracture a fascicle with a fracture stress, σ_U_, of 58.2 MPa (4 month-old age group, Table 2). Numerically, it follows that δσ=2 MPa. The σ_U_ for the 4 month-old age group has a mean value equal to 58.6 MPa with an estimated precision of 4.1 (Table 2), which is about two times larger than the δσ. We can estimate the uncertainty (δE) in the fascicle stiffness, E, using the derived uncertainties in the ε and σ. Numerically, using the mean E=619 MPa from the 4 month-old age group (Table 2), it follows that δE=20 MPa. The estimated precision of the mean E is equal to 58.3 MPa (Table 2); this is about 2.5 times larger than the δE.

To continue the technical discussion on the estimation of the bias error, for the measurement of the fibril structural quantities, we note that the spatial calibration of the TEM employed a grating with 2176 lines per mm; numerically this results in an uncertainty of 0.5 nm. For the purpose of illustration, the uncertainty in the fibril diameter (δD) for a fibril with D=400 nm (e.g. the maximum diameter observed in the 4 month-old age group [strucprop.xls, Data Citation 1]) is equal to 0.5 nm; correspondingly the fibril yields a cross-sectional area (a_f_) of 1×10^5^ nm^2^ with an uncertainty δa_f_=3×10^2^ nm^2^. To estimate the uncertainty (δρ) in the collagen area fraction, ρ, we consider the TEM maximum field-of-view to be 5 μm by 4 μm with a respective uncertainty of about 0.5 nm (following the uncertainty arising from the grating calibration). Numerically, the area of the field-of-view equals 2×10^7^ nm^2^, with an uncertainty of 3×10^3^ nm^2^. By considering the respective uncertainties in the δa_f_ and the area of the field-of-view, numerically, for a given mean ρ=0.85 (i.e. 4 month-old age group, Table 2), it follows that δρ=2x10^-3^. The estimated precision of the mean ρ is equal to 0.01 (Table 2); this is five times larger than the δρ. The routine fixation/dehydration and resin infiltration/polymerisation procedures for electron microscopy inevitably lead to changes in the tissue architecture, involving differential changes in the interfibrillar matrix and the fibrils^42^. According to a synchrotron X-ray study of the changes occurring in the corneal stroma during processing for TEM, the interfibrillar spacing could shrink by 3.6 nm (5.6%), while the intermolecular spacing, which parameterizes the packing of molecules in the transverse section of the fibril, could expand by 0.2 nm (12.8%)^42^. The former was attributed to resin polymerisation, where the epoxy resin could have shrunk during thermal polymerization; the latter was attributed to the post-fixation agent, osmium tetroxide, which could attempt to form intermolecular cross-links, and this in turn could disrupt the hydrogen bonds (including those present in the water molecules) bridging between the α-chains in the collagen molecule^42^. How then would the changes in the interfibrillar spacing and intermolecular spacing interact to affect the absolute value of the D and ρ? To the best of our knowledge, we are not aware of any data on the relative importance of shrinkage at the length scale of the fibril and interfibrillar matrix. Shrinkage in the interfibrillar matrix, in parallel with an increase in intermolecular spacing, would tend to overestimate ρ. How this could bias the determination of age-related changes in ρ as age increases is not clear. We recommend interpreting the ρ and D findings conservatively.

## Usage Notes

There are several predictable uses for these datasets. Firstly, the load-displacement data, as well as the stress-strain data, offer opportunities for further studies to predict how the different ECM structural components at different length-scale contribute to the mechanical response of the tissue, based on multiple length-scale modelling approaches. Secondly, these datasets can be used in comparison with similar data on several other tissues from different parts of the mouse as a model for ageing studies, to aid further understanding of how different tissues respond to the ageing process.

## Additional information

**How to cite this article**: Goh, K. L. *et al*. Age-related dataset on the mechanical properties and collagen fibril structure of tendons from a murine model. *Sci. Data* 5:180140 doi: 10.1038/sdata.2018.140 (2018).

**Publisher’s note**: Springer Nature remains neutral with regard to jurisdictional claims in published maps and institutional affiliations.

## Supplementary Material



## Figures and Tables

**Table 1:** Samples for mechanical testing and transmission electron microscopy (TEM).

**Source**	**P**	**Samples**	**P**	**Data 1**	**P**	**Data 2**	**P**	**Data 3**	**P**	**Data 4**
01 M	P1	mtt01_1	P2	mtt01.zip	P3	mechprop.xls	P4	Scan001 -046.zip	P5	strucprop.xls
01 M		mtt01_2								
01 M		mtt01_3								
02 M		mtt02_1		mtt02.zip				Scan049 -076.zip		
02 M		mtt02_2								
02 M		mtt02_3								
04 M		mtt04_1		mtt04.zip				Scan079 -114.zip		
04 M		mtt04_2								
04 M		mtt04_3								
11 M		mtt11_1		mtt11.zip				Scan115 -125.zip		
11 M		mtt11_2								
11 M		mtt11_3								
11 M		mtt11_4								
23 M		mtt23_1		mtt23.zip				Scan127 -138.zip		
23 M		mtt23_2								
23 M		mtt23_3								
29 M		mtt29_1		mtt29.zip				Scan139 -153.zip		
29 M		mtt29_2								
29 M		mtt29_3								
29 M		mtt29_4								
31 M		mtt31_1		mtt31.zip				Scan154 -168.zip		
31 M		mtt31_2								
31 M		mtt31_3								
31 M		mtt31_4								
35 M		mtt35_1		mtt35.zip				Scan169 -192.zip		
35 M		mtt35_2								
35 M		mtt35_3								
35 M		mtt35_4								
Notes: The Source is classified according to the age group id. The age group ids 01 M, 02 M, 04 M, 11 M, 23 M, 29 M, 31 M and 35 M correspond to 1.6, 2.6, 4.0, 11.5, 23.0, 29.0 31.5 and 35.3 months, respectively. The 'Sample' column indicates the mouse replicates for each age group. P1 represents protocol 1, which involved fascicle extractions from the individual mouse. P2 and P3 represent the mechanical testing protocols 2 and 3, respectively, which involved micromechanical tests and the determination of the mechanical properties. P4 and P5 represent the TEM protocols 4 and 5, respectively, which involved TEM examination and determination of the structural properties from the TEM images. After obtaining the fascicles (the 'Sample' column), P2 was executed to derive Data 1; thereafter P3 was applied to Data 1 to derive Data 2. Similarly, following the 'Sample' column again, P4 was executed to derive Data 3, which was then used by P5 to derive Data 4. 'mtt' stands for mouse tail tendon. The age group id follows after 'mtt'; the last number after the age group id refers to the sample id number.										

**Table 2:** Descriptive statistics of the mechanical and structural properties of the mouse tail tendon.

**Age group**	**1.6 Months**	**2.6 Months**	**4.0 Months**	**11.5 Months**	**23.0 Months**	**29.0 Months**	**31.5 Months**	**35.3 Months**
N	3	3	3	4	3	4	4	4
ρ	0.56±0.01	0.79±0.01	0.85±0.01	0.78±0.01	0.76±0.03	0.81±0.02	0.78±0.02	0.76±0.02
D_D1_ (nm)	104±32	106±40	260±60	111±30	65±22	56±9	50±12	42±9
D_D2_ (nm)	178±68	201±46	340±20	250±50	240±61	220±82	230±85	214±78
E (MPa)	376.9±61.3	574.4±52.2	619.8±58.3	626.2±94.9	566.2±47.9	624.4±14.0	575.8±66.2	465.2±46.7
σ_Y_(MPa)	11.9±1.9	19.6±1.6	21.2±1.5	28.0±8.9	26.5±2.2	27.6±0.9	23.7±1.6	20.0±0.3
σ_U_ (MPa)	26.6±3.2	42.6±3.4	58.6±4.1	62.4±7.4	58.6±5.4	60.3±3.7	51.6±4.4	45.6±1.7
u_Y_ (MPa)	0.23±0.06	0.38±0.04	0.44±0.03	0.80±0.31	0.78±0.07	0.74±0.03	0.63±0.04	0.57±0.07
u_F_ (MPa)	3.50±0.39	5.90±0.54	8.48±0.54	7.16±1.40	7.42±0.85	7.72±0.88	5.98±0.41	5.74±0.30
u_0_ (MPa)	3.74±0.42	6.28±0.42	8.93±0.51	7.92±1.62	8.20±0.91	8.45±0.91	6.61±0.42	6.32±0.24
u_Y_/σ_Y_	0.0194±0.0023	0.0184±0.0010	0.0190±0.0015	0.0249±0.0045	0.0281±0.0010	0.0258±0.0003	0.0262±0.0023	0.0255±0.0025
u_F_/σ_U_	0.1249±0.0030	0.1322±0.0074	0.1460±0.0041	0.1174±0.0216	0.1235±0.0041	0.1312±0.0087	0.1179±0.0040	0.1257±0.0064
Note: N refers to the number of mice. The number before the±sign is a mean value. The number after the±sign in the entries of D_D1_ and D_D2_ is a standard deviation (SD) but the number after the±sign in the other entries is a standard error (SEM).								
